# Predicting Stroke Outcomes Using Ankle-Brachial Index and Inter-Ankle Blood Pressure Difference

**DOI:** 10.3390/jcm9041125

**Published:** 2020-04-15

**Authors:** Minho Han, Young Dae Kim, Jin Kyo Choi, Junghye Choi, Jimin Ha, Eunjeong Park, Jinkwon Kim, Tae-Jin Song, Ji Hoe Heo, Hyo Suk Nam

**Affiliations:** 1 Department of Neurology, Yonsei University College of Medicine, Seoul 03722, Korea; 2 Integrative Research Center for Cerebrovascular and Cardiovascular Diseases, Seoul 03722, Korea; 3 Cardiovascular Research Institute, Yonsei University College of Medicine, Seoul 03722, Korea; 4 Department of Neurology, Yongin Severance Hospital, Yonsei University College of Medicine, Yongin-si 16995, Korea; 5 Department of Neurology, Seoul Hospital, Ewha Womans University College of Medicine, Seoul 07804, Korea

**Keywords:** ankle-brachial index difference, inter-ankle blood pressure difference, stroke, peripheral artery disease, outcome

## Abstract

Background: This study investigated the association of high ankle-brachial index difference (ABID) and systolic inter-ankle blood pressure difference (IAND) with short- and long-term outcomes in acute ischemic stroke patients without peripheral artery disease (PAD). Methods: Consecutive patients with acute ischemic stroke who underwent ankle-brachial index (ABI) measurement were enrolled. ABID was calculated as |right ABI-left ABI|. IAND and systolic inter-arm blood pressure difference (IAD) were calculated as |right systolic blood pressure – left systolic blood pressure|. Poor functional outcome was defined as modified Rankin Scale score ≥3 at 3 months. Major adverse cardiovascular events (MACEs) were defined as stroke recurrence, myocardial infarction, or death. Results: A total of 2901 patients were enrolled and followed up for a median of 3.1 (interquartile range, 1.6–4.7) years. Among them, 2643 (84.9%) patients did not have PAD. In the logistic regression analysis, ABID ≥ 0.15 and IAND ≥ 15 mmHg were independently associated with poor functional outcome (odds ratio (OR), 1.970, 95% confidence interval (CI), 1.175‒3.302; OR, 1.665, 95% CI, 1.188‒2.334, respectively). In Cox regression analysis, ABID ≥0.15 and IAND ≥ 15 mmHg were independently associated with MACEs (hazard ratio (HR), 1.514, 95% CI, 1.058‒2.166; HR, 1.343, 95% CI, 1.051‒1.716, respectively) and all-cause mortality (HR, 1.524, 95% CI, 1.039‒2.235; HR, 1.516, 95% CI, 1.164‒1.973, respectively) in patients without PAD. Conclusion: High ABID and IAND are associated with poor short-term outcomes, long-term MACE occurrence, and all-cause mortality in acute ischemic stroke without PAD.

## 1. Introduction

Blood pressure (BP) ratios and differences between the four limbs can be simultaneously obtained and calculated with ankle-brachial index (ABI) measurement [1]. Among the ratios and differences, ABI difference (ABID), systolic inter-ankle blood pressure difference (IAND), and systolic inter-arm BP difference (IAD) have been reported to be useful in predicting the prognosis in patients with cardiovascular disease, high-risk populations, and the general population [2,3].

Lower extremity peripheral artery disease (PAD) is defined by a low ABI, calculated by dividing the ankle systolic BP by the arm systolic BP. ABI has high specificity and sensitivity for the diagnosis of PAD [4], and ABI may also provide information beyond PAD. A previous study showed that ABID ≥ 0.15 was an independent risk factor for overall mortality in patients undergoing hemodialysis [5]. However, the prognostic value of ABID in patients with ischemic stroke remains uncertain.

IAD is strongly associated with increased cardiovascular and all-cause mortality [6]. Previous studies showed that IAND provided additional information to estimate stroke incidence and cardiovascular mortality beyond IAD [1,3]. To the best of our knowledge, no study has reported the prognostic impact of IAND on the outcomes of patients with acute ischemic stroke.

A previous study showed that the prevalence of PAD in patients with ischemic stroke was 32% and the rate of asymptomatic PAD in patients with stroke was 68% [7]. Another study showed that stroke patients with asymptomatic PAD had an increased risk of recurrent vascular events, including stroke [8]. Therefore, the prognostic significance needs to be separately assessed in ischemic stroke patients without PAD.

In this regard, we hypothesized that ABID and IAND are associated with poor short-term functional outcomes, major adverse cardiovascular events (MACEs), and all-cause mortality in patients with acute ischemic stroke. Whether the prognostic values of these parameters are valid in acute ischemic stroke patients without PAD was also investigated.

## 2. Materials and Methods

### 2.1. Patients and Evaluation

A hospital-based, retrospective observational study using prospectively collected stroke registry data was conducted. The Yonsei Stroke Registry collected the data of patients with acute cerebral infarction or transient ischemic attack (TIA) who presented to the emergency department within 7 days of symptom onset between January 1, 2007 and June 30, 2013 [9]. Acute cerebral infarction was defined as sudden onset of acute neurological deficits of presumed vascular etiology lasting 24 h or evidence of acute infarction on brain computed tomography (CT) or magnetic resonance imaging (MRI). TIA was diagnosed when a patient had transient (<24 h) neurologic dysfunction of vascular origin and did not show acute lesions on CT or MRI. Among these candidates, only patients with available four-limb BPs measured by ABI examination and a cerebral angiographic evaluation using either CT angiography, MR angiography, or digital subtraction angiography performed during the admission period were included. Patients were treated by standard treatment protocols based on the guidelines for acute ischemic stroke [10,11,12,13]. Stroke classifications were determined during weekly conferences. Based on a consensus of three stroke neurologists, stroke subtypes were classified according to the Trial of ORG 10172 in Acute Stroke Treatment (TOAST) classification [14]. 

### 2.2. Demographic Characteristics and Risk Factors

We collected data on baseline characteristics, including sex, age, and neurological deficit (National Institutes of Health Stroke Scale (NIHSS) score) upon admission; presence of risk factors; and laboratory data (glucose, high-density lipoprotein (HDL), and low-density lipoprotein (LDL)). Hypertension was defined as resting systolic blood pressure (SBP) of ≥140 mmHg or diastolic blood pressure (DBP) of ≥90 mmHg after repeated measurements during hospitalization or currently taking antihypertensive medication. Diabetes mellitus was defined as fasting plasma glucose levels of ≥7 mmol/L or taking an oral hypoglycemic agent or insulin. Current smoking was defined as having smoked a cigarette within 1 year prior to admission. Congestive heart failure was determined from the history of heart failure diagnosis, treatment with loop diuretics, and ejection fraction of ≤35% on echocardiography. Coronary artery disease (CAD) was diagnosed when a patient had a previous history of CAD (acute myocardial infarction, unstable angina, coronary artery bypass graft, or percutaneous coronary artery stent/angioplasty) or the presence of significant stenosis (≥50%) in any of the three main coronary arteries on multi-slice CT coronary angiography upon admission. Cerebral artery atherosclerosis (CAA) was defined as occlusion or significant stenosis (≥50%) of any intracranial or extracranial cerebral artery. PAD was determined if a patient had an ABI of <0.9 or a history of angiographically confirmed PAD.

### 2.3. ABI and Brachial-Ankle Pulse Wave Velocity Measurement 

ABI and brachial-ankle pulse wave velocity (baPWV) were measured in the supine position using an automatic device (VP-1000; Colin Co., Ltd., Komaki, Japan), which has been validated previously [6,15]. This device automatically and simultaneously measures four-limb pulse wave forms and BP using the oscillometric method. Right ABI was calculated by the ratio of the right ankle SBP divided by the higher SBP of the arms. Left ABI was calculated by the ratio of the left ankle SBP divided by the higher SBP of the arms. ABID was calculated as |right ABI-left ABI|. IAND was extracted as BPs from both legs and calculated as |right ankle SBP-left ankle SBP|. IAD was extracted as BPs from arms and calculated as |right brachial SBP-left brachial SBP|. BaPWV on each side was automatically calculated as the transmission distance divided by the transmission time and expressed in centimeters per second. Transmission distance from the arm to each ankle was automatically calculated according to the patient’s height. Transmission time was defined as the time interval between the initial increase of brachial and tibial waveforms. The higher values of baPWV on both sides were used for analysis.

### 2.4. Follow-Up and Outcome Measures

Patients were followed up in the outpatient clinic or by a structured telephone interview at 3 months and yearly after discharge. Short-term functional outcomes at 3 months were determined by a structured interview using the modified Rankin Scale (mRS). Poor outcome was defined as an mRS of ≥3. Deaths among participants from January 1, 2007 to December 31, 2013, were confirmed by matching the information in the death records and identification numbers assigned to the participants at birth [16]. We obtained data for the date and causes of death from the Korean National Statistical Office, which were identified based on death certificates. MACEs were defined as any stroke recurrence, myocardial infarction occurrence, or death.

### 2.5. Statistical Analysis

SPSS for Windows (version 23, SPSS, Chicago, IL, USA) was used for the statistical analysis. Intergroup statistical analyses were performed to compare the demographic characteristics and risk factors in the whole study population. The statistical significance of intergroup differences was assessed using the χ^2^ or Fisher’s exact test for categorical variables and independent two-sample *t*-test or Mann–Whitney *U*-test for continuous variables. Data were expressed as means ± standard deviations or medians (interquartile ranges (IQRs)) for continuous variables and numbers (%) for categorical variables. Cutoff values for IAND and IAD were based on those used in the previous study [3]. In elderly people, IAND of ≥15 mmHg and IAD of ≥15 mmHg were cutoff values that could predict mortality [3]. The cutoff value of ABID of ≥0.15 mmHg was based on a study wherein ABID predicted the mortality of patients with chronic hemodialysis [5]. Multivariable logistic regression analysis was performed after adjusting for sex, age, cardiovascular risk factors (hypertension, diabetes mellitus, hypercholesterolemia, current smoking, congestive heart failure, CAD, CAA, and PAD), and variables that exhibited a *p* value of <0.05 in the univariate analysis, to investigate the association of ABID, IAND, or IAD with short-term functional outcomes. Survival curves were generated according to the Kaplan–Meier method and compared using the log-rank test. Multivariable Cox proportional hazard regression was performed to determine independent factors associated with survival after an ischemic stroke. Subgroup analysis was also performed to confirm that the associations between short- and long-term outcomes and BP differences were valid in patients without PAD. We analyzed the diastolic IAND and diastolic IAD separately as supplemental data. All *P* values were two-tailed, and differences were considered significant at *p* < 0.05.

### 2.6. Standard Protocol Approval, Registration, and Patient Consent

The Institutional Review Board of Severance Hospital, Yonsei University Health System, approved this study and waived the need for informed consent because of the retrospective design and observational nature of this study (approval date: 2020-01-16; approval number: 4-2019-1196).

### 2.7. Data availability Statement

De-identified participant data are available upon reasonable request. 

## 3. Results

### 3.1. Patient Demographic and Clinical Characteristics 

**Figure 1:**
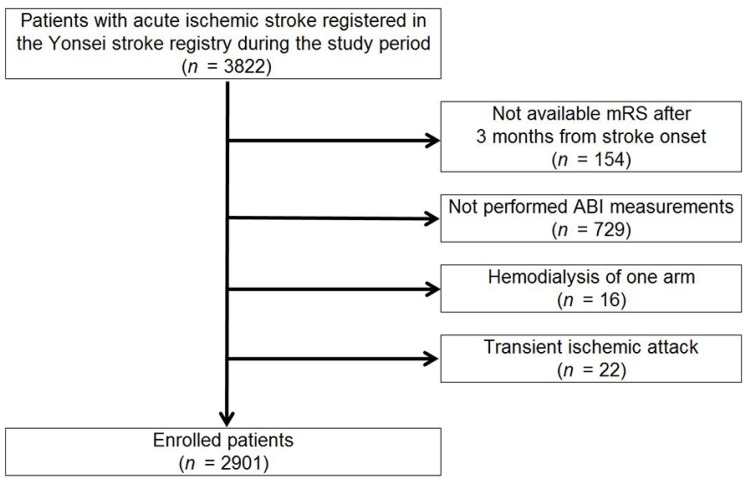
Flowchart of participants according to inclusion and exclusion criteria. ABI, ankle brachial index; mRS; modified Rankin Scale score.

A total of 3822 patients with acute ischemic stroke or TIA were recruited during the study period. After exclusions (follow-up loss (*n* = 154), no ABI measurements (*n* = 729), hemodialysis of one arm (*n* = 16), and TIA (*n* = 22)), 2901 patients were finally enrolled in this study (Figure 1).

A total of 258 (8.9%) patients had PAD. The mean age was 65.4 ± 12.2 years, and 61.8% were men. Among them, 582 (20.1%) had poor outcomes (Table 1). Compared with patients with good outcomes, those with poor outcomes were older, were more likely to be women, had more severe initial stroke severity, were less likely to be current smokers, and were more likely to have CAA, PAD, and a stroke subtype of large artery atherosclerosis (all *p* values <0.05). For four-limb BP profiles, both ankle SBP and ABI were lower in patients with poor outcomes than in those with good outcomes (all *p* values <0.001). All BP differences including ABID, IAND, and IAD were higher in patients with poor outcomes than in those with good outcomes (all *p* values <0.001). Compared with the included patients, excluded patients were older, were more likely to be women, had higher NIHSS score, were more likely to have congestive heart failure, and were less likely to be current smokers or have CAD (Supplementary Table S1).

In all study patients, 236 (8.1%) patients showed ABID ≥0.15, 450 (15.5%) had IAND ≥15 mmHg, and 116 (4.0%) had IAD ≥15 mmHg. Among atherosclerotic diseases, CAA and PAD were independent determinants of ABID ≥0.15 (CAA: odds ratio (OR), 1.718, 95% confidence interval (CI), 1.211‒2.437; PAD: OR, 22.124, 95% CI, 15.844‒30.894) and IAND ≥15 mmHg (CAA: OR, 1.646, 95% CI, 1.281‒2.114; PAD: OR, 13.328, 95% CI, 9.876‒17.987). However, only PAD was an independent determinant of IAD ≥15 mmHg (PAD: OR, 3.044, 95% CI, 1.890‒4.904) (Table 2). 

ABID ≥0.15 and IAND ≥15 mmHg were more likely to have ABI >1.30 (all *p* values <0.001), but not IAD ≥15 mmHg (Table 3). BaPWVs were well correlated with ABID, IAND, and IAD (with ABID, *r* = 0.139, *p* < 0.001; with IAND, *r* = 0.207, *p* < 0.001; and with IAD, *r* = 0.121, *p* < 0.001) (Supplementary Table S2).

### 3.2. Poor Functional Outcome

All patients with (*n* = 2901) and without PAD (*n* = 2643) were separately analyzed. In all study patients, poor outcome was independently associated with ABID (OR, 5.289, 95% CI, 1.723‒16.236) and cutoff of ABID ≥0.15 (OR, 1.920, 95% CI, 1.361‒2.708). Poor outcome was also independently associated with IAND (OR, 1.015, 95% CI, 1.007‒1.023) and cutoff of IAND ≥15 mmHg (OR, 1.818, 95% CI, 1.389‒2.381). In patients without PAD, the cutoff of ABID ≥0.15 was independently associated with poor outcomes (OR, 1.970, 95% CI, 1.175‒3.302). IAND and cutoff of IAND ≥15 mmHg were also independently associated with poor outcomes (IAND: OR, 1.025, 95% CI, 1.009‒1.041; IAND ≥15 mmHg: OR, 1.665, 95% CI, 1.188‒2.334). Conversely, IAD ≥15 mmHg was associated with poor outcomes in the whole population (OR, 1.623, 95% CI, 1.011‒2.605) but was not associated with poor outcomes in patients without PAD (Table 4).

### 3.3. All-Cause Mortality and MACEs

**Figure 2:**
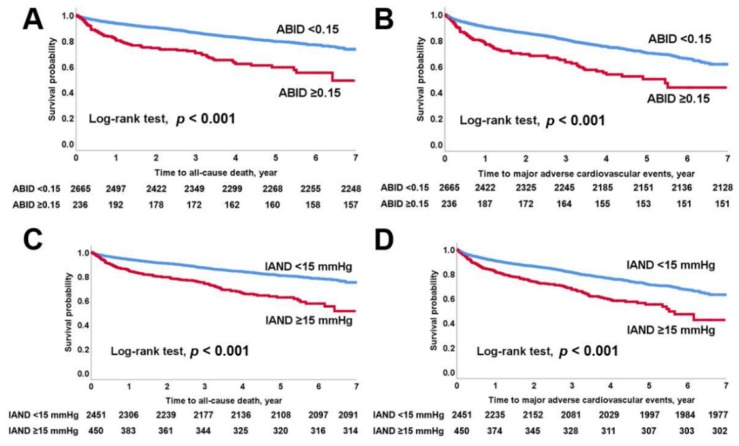
Kaplan–Meier survival analysis. (**A**) All-cause mortality; (**B**) major adverse cardiovascular event according to ABID ≥0.15. (**C**) All-cause mortality; (**D**) major adverse cardiovascular event according to IAND ≥15 mmHg. ABID; ankle-brachial index difference; IAND, systolic inter-ankle blood pressure difference.

Study patients were followed up for a median of 3.1 (IQR, 1.6–4.7) years. A total of 622 patients had MACEs (21.4%) including 496 all-cause deaths (17.1%) during the study period. In Kaplan–Meier survival curves (Figure 2), higher all-cause mortality and MACEs (log-rank test; *p* < 0.001) were found in patients with ABID ≥0.15 or IAND ≥15 mmHg (log-rank test; all *p* < 0.001). Higher all-cause mortality (log-rank test; *p* = 0.007) and MACEs (log-rank test; *p* = 0.008) were also found in patients with IAD ≥15 mmHg. 

In multivariable Cox regression analysis, ABID and ABID ≥0.15 were independently associated with all-cause mortality (ABID: hazard ratio (HR), 6.221, 95% CI, 2.973‒13.018; ABID ≥0.15: HR, 1.567, 95% CI, 1.223‒2.009) and MACEs (ABID: HR, 3.926, 95% CI, 1.906‒8.087; ABID ≥0.15: HR, 1.416, 95% CI, 1.117‒1.794). IAND and IAND ≥15 mmHg were also independently associated with all-cause mortality (IAND: HR, 1.013, 95% CI, 1.007‒1.019; IAND ≥15 mmHg: HR, 1.616, 95% CI, 1.317‒1.982) and MACEs (IAND: HR, 1.010, 95% CI, 1.005‒1.015; IAND ≥15 mmHg: HR, 1.380, 95% CI, 1.139‒1.672). 

In patients without PAD, ABID and ABID ≥0.15 were independently associated with all-cause mortality (ABID: HR, 9.221, 95% CI, 3.013‒28.220; ABID ≥0.15: HR, 1.524, 95% CI, 1.039‒2.235) and MACEs (ABID: HR, 6.605, 95% CI, 2.281‒19.124; ABID ≥0.15: HR, 1.514, 95% CI, 1.058‒2.166). IAND and IAND ≥15 mmHg were also independently associated with all-cause mortality (IAND: HR, 1.017, 95% CI, 1.004‒1.030; IAND ≥15 mmHg: HR, 1.516, 95% CI, 1.164‒1.973) and MACEs (IAND: HR, 1.015, 95% CI, 1.004‒1.027; IAND ≥15 mmHg: HR, 1.343, 95% CI, 1.051‒1.716). Meanwhile, IAD was associated only with the long-term occurrence of MACEs in all patients (HR, 1.010, 95% CI, 1.001‒1.019), but not in those without PAD (HR, 1.006, 95% CI, 0.993‒1.018, *p* = 0.374) (Table 5).

## 4. Discussion

We demonstrated that higher ABID and IAND were independently associated with poor short-term functional outcomes, long-term MACE occurrence, and all-cause mortality in patients with acute ischemic stroke. In particular, higher ABID and IAND had prognostic effects even in patients without PAD. Meanwhile, IAD was associated with poor short-term outcomes and MACEs in all patients, but not in those without PAD. These findings suggest that higher ABID and IAND have prognostic value for both poor short- and long-term outcomes of acute ischemic stroke and are more sensitive than IAD for predicting outcomes in acute ischemic stroke patients without PAD.

Primarily, increased ABID and IAND are attributable to the presence of PAD [17]. PAD affects approximately 200 million people worldwide and is the third most common cause of atherosclerotic cardiovascular death after CAD and stroke [18]. Traditional cardiovascular risk factors (smoking, hypertension, diabetes mellitus, and hypercholesterolemia) and advanced aging are important determinants of PAD. Therefore, patients with PAD often have concomitant atherosclerosis on the cerebral and coronary artery. In the Reduction of Atherothrombosis for Continued Health registry involving 44 countries worldwide, 39% of patients with PAD had CAD, 10% had cerebral artery disease, and 13% had both [19]. Accumulated systemic atherosclerosis worsens stroke prognosis [20]. Among the atherosclerotic burdens, ABID and IAND were associated with CAA and PAD. In contrast, IAD was only associated with PAD. It can be assumed that stroke patients with large ABID and IAND are more likely to have additional cerebral atherosclerotic burden and may have a poorer prognosis.

To the best of our knowledge, no study has previously evaluated ABID as a prognosis predictor in patients with acute ischemic stroke. ABI measurement is a well-established method to identify patients with PAD. Low ABI is commonly defined as ABI <0.9 and provides good sensitivity (80%) and excellent specificity (95%) to detect PAD [4]. ABI is also associated with poor initial stroke severity [21] and predicts poor prognosis and mortality in patients with stroke [2]. However, several previous studies have shown that low ABI was not sensitive enough to detect asymptomatic PAD in the general population [22]. To detect PAD and predict stroke prognosis accurately, novel parameters besides ABI should be developed. We found that ABID and IAND were independent and strong predictors of MACEs and all-cause mortality in patients with acute ischemic stroke. Interestingly, ABID and IAND remained to be significantly associated with poor short- and long-term outcomes in patients without PAD. This finding suggests that ABID and IAND may provide additional information for patients with subclinical or mild PAD. The strength of ABID might be related to the consideration of IAND and arm BP simultaneously. In addition, ABID and IAND can be obtained and easily calculated during ABI measurement in routine clinical practice.

In patients with stroke, IAD has been demonstrated to be associated with recurrent stroke [23], poor prognosis [24], and mortality [6]. However, some patients undergo dialysis with one arm because of end-stage renal disease, making it difficult to measure IAD. In addition, IAD (and ABI) may be “pseudonormal” when a patient has severe stenosis in both arms and in one leg. In contrast, IAND can be calculated without BP measurement in the arm and provide consistent data [3]. Several studies showed the increased usefulness of IAND and ABI relative to IAD. One study showed that IAND could better predict both overall and cardiovascular mortality than IAD in elderly patients [3]. ABI exhibits better association with cardiovascular outcomes than IAD in patients with type 2 diabetes [25]. However, no study has reported the comparison between IAND and IAD in patients with acute ischemic stroke. 

Because lower limbs are more prone to be affected by PAD than upper limbs, IAND could be a better predictor of PAD than IAD [26]. High IAND was associated with increased left ventricular mass index [27] and arterial stiffness [28] and also predicted mortality in the elderly people [3]. Similarly, large ABID provided the prognostic value for mortality in patients undergoing chronic hemodialysis [5]. These findings suggest that the cardiovascular risk was higher in patients with lower extremity PAD than in those with upper extremity PAD [29]. Therefore, the circulatory burden assumed from the heart to the ankles may be greater than that from the heart to the arms. 

Endothelial dysfunction [26], calcification burden [30], and arterial stiffness [27] are more frequent in the lower extremities than in the upper extremities. The degree of endothelial dysfunction in leg circulation is related to PAD severity. Endothelial dysfunction of leg circulation may occur before the impairment of forearm circulation in PAD [26]. Our data showed that high ABID and IAND were more likely to have ABI >1.30 than IAD. High ABI (i.e., ABI >1.30) is generally believed to occur because of medial arterial calcification and may be a marker for vascular stiffness [4]. High ABI was associated with an increase in both overall and cardiovascular mortality in patients with chronic kidney disease undergoing hemodialysis [31] and in the general population [32]. PWV and ABI are both atherosclerotic markers. ABI reflects stenosis or peripheral artery obstruction, whereas PWV represents arterial stiffness [5]. ABID, IAND, and IAD was positively correlated with baPWV. The correlation coefficient was highest in IAND, followed by ABID and IAD. In patients undergoing hemodialysis, high PWV and low ABI are significantly associated with mortality [33]. Therefore, ABID and IAND could be more influenced by endothelial dysfunction, systemic atherosclerosis, calcification burden, and arterial stiffness than IAD, which may be related to more frequent PAD in the lower extremities [25,26,28,29].

This study has several limitations. First, radiological studies to detect atherosclerosis in the lower extremities were not routinely performed. A correlation study between apparent atherosclerosis and IAND or ABID might be helpful for better understanding [4,17]. Second, multiple, automatic, and simultaneous assessments are recommended for accurate BP difference measurement rather than single, manual, and sequential evaluation methods [34]. We used an automatic and simultaneous measurement device, but BP difference was investigated only once during the ABI measurement, and additional follow-up data were limited. Third, BP differences in this study focused on SBP, rather than DBP. Additional analysis was performed with DBP data, which found that the prognostic effect of DBP was not different from that of SBP (Supplementary Tables S3 and S4). Fourth, our findings may not be generalized to other populations or cohorts because our study population is limited to Korean patients. Fifth, the stroke standard treatment guidelines were updated and changed several times during the study period. Lastly, a total of 921 patients were excluded from the analysis. Among them, patients who did not undergo ABI measurements were mostly excluded. Therefore, the possibility of selection bias exists because of the retrospective study design; however, consecutive patients were included, and a relatively large sample size was analyzed.

## 5. Conclusions

This study suggests that high ABID and IAND are associated with poor short-term outcomes, long-term MACE occurrence, and all-cause mortality in patients with acute ischemic stroke. In addition, ABID and IAND predict post-stroke outcomes, even in patients without PAD. Therefore, ABID and IAND can be simple and reliable methods for identifying patients with an increased risk of poor short- and long-term outcomes in acute ischemic stroke.

## Figures and Tables

**Table 1:** Patient demographic and clinical characteristics.

	Total	Good Outcomes (mRS of 0‒2; *n* = 2319)	Poor Outcomes (mRS of 3‒6; *n* = 582)	*p* Value
	(*n* = 2901)			
Age, y	65.4 ± 12.2	64.0 ± 12.0	71.0 ± 11.4	<0.001
Men	1793 (61.8)	1489 (64.2)	304 (52.2)	<0.001
NIHSS score at admission	3.0 (1.0, 6.0)	2.0 (1.0, 4.0)	8.0 (4.0, 15.0)	<0.001
**Risk factors**				
Hypertension	2164 (74.6)	1712 (73.8)	452 (77.7)	0.057
Diabetes mellitus	920 (31.7)	728 (31.4)	192 (33.0)	0.459
Hypercholesterolemia	622 (21.4)	486 (21.0)	136 (23.4)	0.205
Current smoking	717 (24.7)	622 (26.8)	95 (16.3)	<0.001
Congestive heart failure	119 (4.1)	92 (4.0)	27 (4.6)	0.465
Coronary artery disease	686 (23.6)	549 (23.7)	137 (23.5)	0.946
Cerebral artery atherosclerosis	1727 (59.5)	1292 (55.7)	435 (74.7)	<0.001
Peripheral artery disease	258 (8.9)	152 (6.6)	106 (18.2)	<0.001
**Laboratory findings**				
Glucose, mg/dL	143.5 ± 63.9	142.7 ± 63.2	1468 ± 66.0	0.168
HDL, mg/dL	42.8 ± 11.0	42.6 ± 10.8	43.4 ± 11.6	0.127
LDL, mg/dL	114.5 ± 38.6	114.7 ± 37.5	113.8 ± 42.5	0.651
**Stroke subtype**				
LAA	587 (20.2)	440 (19.0)	147 (25.3)	<0.001
CE	754 (26.0)	600 (25.9)	154 (26.5)	
SVO	261 (9.0)	232 (10.0)	29 (5.0)	
OC	72 (2.5)	58 (2.5)	14 (2.4)	
UE	1227 (42.3)	989 (42.6)	238 (40.9)	
**Arm BP, mmHg**				
Right SBP	146.3 ± 23.5	146.7 ± 23.2	145.1 ± 24.6	0.147
Left SBP	145.3 ± 23.8	145.6 ± 23.6	144.0 ± 24.6	0.129
IAD	4.90 ± 6.51	4.71 ± 6.45	5.77 ± 7.15	0.001
**Ankle BP, mmHg**				
Right SBP	164.5 ± 31.3	166.3 ± 30.1	157.7 ± 35.1	<0.001
Left SBP	163.6 ± 31.3	165.2 ± 30.4	157.2 ± 34.6	<0.001
IAND	9.23 ± 11.94	8.42 ± 10.82	12.65 ± 15.87	<0.001
**ABI**				
Right ABI	1.111 ± 0.132	1.122 ± 0.118	1.071 ± 0.170	<0.001
Left ABI	1.105 ± 0.130	1.114 ± 0.118	1.069 ± 0.171	<0.001
ABID	0.063 ± 0.083	0.058 ± 0.077	0.086 ± 0.104	<0.001
Right ABI >1.30	58 (2.0)	44 (1.9)	14 (2.4)	0.434
Left ABI >1.30	44 (1.5)	31 (1.3)	13 (2.2)	0.113
Both ABI >1.30	18 (0.6)	14 (0.6)	4 (0.7)	0.818

Data are expressed as means ± standard deviations, medians [interquartile ranges], or numbers (%). ABI, ankle brachial index; ABID, ankle brachial index difference; BP, blood pressure; CE, cardioembolism; DBP, diastolic blood pressure; HDL, high density lipoprotein; IAD, systolic inter-arm blood pressure difference; IAND, systolic inter-ankle blood pressure difference; LAA, large artery atherosclerosis; LDL, low density lipoprotein; mRS, modified Rankin Scale; NIHSS, National Institutes of Health Stroke Scale; OC, other cause; SBP, systolic blood pressure; SVO, small vessel occlusion; and UE, undetermined etiology.

**Table 2:** Determinants of IAD ≥15 mmHg, IAND ≥15 mmHg, and ABID ≥0.15.

	ABID ≥0.15		IAND ≥15 mmHg		IAD ≥15 mmHg	
	OR (95% CI)	*p* value *	OR (95% CI)	*p* value *	OR (95% CI)	*p* value *
CAD	1.290 (0.954‒1.745)	0.098	0.957 (0.752‒1.217)	0.718	0.912 (0.580‒1.434)	0.689
CAA	1.718 (1.211‒2.437)	0.002	1.646 (1.281‒2.114)	<0.001	1.451 (0.926‒2.274)	0.104
PAD	22.124 (15.844‒30.894)	<0.001	13.328 (9.876‒17.987)	<0.001	3.044 (1.890‒4.904)	<0.001

Data were derived from the multivariable logistic regression analysis. ABID, ankle brachial index difference; CAD, coronary artery disease; CAA, cerebral artery atherosclerosis; CI, confidence interval; IAD, systolic inter-arm blood pressure difference; IAND, systolic inter-ankle blood pressure difference; NIHSS, National Institutes of Health Stroke Scale; OR, odds ratio; and PAD, peripheral artery disease. *adjusted for sex, age, NIHSS score at admission, hypertension, diabetes mellitus, hypercholesterolemia, current smoking, congestive heart failure, and stroke subtype.

**Table 3:** Relationship between IAD, IAND, ABID, and ABI >1.30.

	Right ABI >1.30		Left ABI >1.30		Both ABI >1.30	
	*n* (%)	*p* value	*n* (%)	*p* value	*n* (%)	*p* value
**ABID**						
ABID <0.15	45 (1.7)	0.001	30 (1.1)	<0.001	18 (0.7)	0.392
ABID ≥0.15	13 (5.5)		14 (5.9)		0 (0.0)	
**IAND**						
IAND <15 mmHg	36 (1.5)	<0.001	27 (1.1)	<0.001	16 (0.7)	1.000
IAND ≥15 mmHg	22 (4.9)		17 (3.8)		2 (0.4)	
**IAD**						
IAD <15 mmHg	55 (2.0)	0.503	42 (1.5)	0.695	17 (0.6)	0.521
IAD ≥15 mmHg	3 (2.6)		2 (1.7)		1 (0.9)	

ABI, ankle brachial index; ABID, ankle brachial index difference; IAD, systolic inter-arm blood pressure difference; and IAND, systolic inter-ankle blood pressure difference.

**Table 4:** Predictors of poor outcome at 3 months.

	All Patients (*n* = 2901)		Patients without PAD (*n* = 2643)	
	OR (95% CI)	*p* value*	OR (95% CI)	*p* value*
**ABI**				
ABID	5.289 (1.723‒16.236)	0.004	5.774 (0.948‒35.151)	0.057
ABID ≥0.15	1.920 (1.361‒2.708)	<0.001	1.970 (1.175‒3.302)	0.010
**Ankle BP, mmHg**				
IAND	1.015 (1.007‒1.023)	<0.001	1.025 (1.009‒1.041)	0.002
IAND ≥15 mmHg	1.818 (1.389‒2.381)	<0.001	1.665 (1.188‒2.334)	0.003
**Arm BP, mmHg**				
IAD	1.009 (0.995‒1.024)	0.190	1.009 (0.991‒1.027)	0.329
IAD ≥15 mmHg	1.623 (1.011‒2.605)	0.045	1.337 (0.758‒2.360)	0.316

Data were derived from the multivariable logistic regression analysis. ABI, ankle brachial index; ABID, ankle brachial index difference; BP, blood pressure; CI, confidence interval; IAD, systolic inter-arm blood pressure difference; IAND, systolic inter-ankle blood pressure difference; NIHSS, National Institutes of Health Stroke Scale; OR, odds ratio; and PAD, peripheral artery disease. *adjusted for sex, age, NIHSS score at admission, hypertension, diabetes mellitus, hypercholesterolemia, current smoking, congestive heart failure, coronary artery disease, cerebral artery atherosclerosis, and stroke subtype.

**Table 5:** Predictors of long-term outcome.

	**All Patients (*n* = 2901)**			
	**All-Cause Mortality**		**MACE**	
	HR (95% CI)	*p* value*	HR (95% CI)	*p* value*
**ABI**				
ABID	6.221 (2.973‒13.018)	<0.001	3.926 (1.906‒8.087)	<0.001
ABID ≥0.15	1.567 (1.223‒2.009)	<0.001	1.416 (1.117‒1.794)	0.004
**Ankle BP, mmHg**				
IAND	1.013 (1.007‒1.019)	<0.001	1.010 (1.005‒1.015)	<0.001
IAND ≥15 mmHg	1.616 (1.317‒1.982)	<0.001	1.380 (1.139‒1.672)	0.001
**Arm BP, mmHg**				
IAD	1.009 (0.999‒1.019)	0.068	1.010 (1.001‒1.019)	0.027
IAD ≥15 mmHg	1.176 (0.810‒1.708)	0.395	1.151 (0.820‒1.617)	0.417
	**Patients without PAD (*n* = 2643)**			
	**All-cause mortality**		**MACE**	
	HR (95% CI)	*p* value*	HR (95% CI)	*p* value*
**ABI**				
ABID	9.221 (3.013‒28.220)	<0.001	6.605 (2.281‒19.124)	0.001
ABID ≥0.15	1.524 (1.039‒2.235)	0.031	1.514 (1.058‒2.166)	0.023
**Ankle BP, mmHg**				
IAND	1.017 (1.004‒1.030)	0.010	1.015 (1.004‒1.027)	0.010
IAND ≥15 mmHg	1.516 (1.164‒1.973)	0.002	1.343 (1.051‒1.716)	0.019
**Arm BP, mmHg**				
IAD	1.007 (0.993‒1.021)	0.333	1.006 (0.993‒1.018)	0.374
IAD ≥15 mmHg	1.075 (0.681‒1.697)	0.755	1.032 (0.682‒1.563)	0.881

Data were derived from the cox proportional hazards regression analysis. ABI, ankle brachial index; ABID, ankle brachial index difference; BP, blood pressure; CI, confidence interval; HR, hazard ratio; IAD, systolic inter-arm blood pressure difference; IAND, systolic inter-ankle blood pressure difference; MACE, major adverse cardiovascular event; NIHSS, National Institutes of Health Stroke Scale; and PAD, peripheral artery disease. *adjusted for sex, age, NIHSS score at admission, hypertension, diabetes mellitus, hypercholesterolemia, current smoking, congestive heart failure, coronary artery disease, cerebral artery atherosclerosis, and stroke subtype.

## Supplementary Materials

The following are available online at https://www.mdpi.com/2077-0383/9/4/1125/s1, Table S1. Comparison of acute stroke patients during the study period who were included and excluded in this study, Table S2. Correlations between IAD, IAND, ABID, and baPWV in all patients (*n* = 2901), Table S3. Predictors of short-term outcome, Table S4. Predictors of long-term outcome.
