# Supplementary material for: Predicting Stroke Outcomes Using Ankle-Brachial Index and Inter-Ankle Blood Pressure Difference
Source: J Clin Med. 2020 Apr 15;9(4):1125. doi: 10.3390/jcm9041125 (PMC7231112; doi:10.3390/jcm9041125)
Supplement: Supplementary file 1 [file jcm-09-01125-s001.zip › SUPPLEMENTAL MATERIAL.docx]

**Supplemental Materials**

**Predicting stroke outcomes using ankle-brachial index and inter-ankle blood pressure difference**

**Supplementary Table S1.** Comparison of acute stroke patients during the study period who were included and excluded in this study.

|  | Included  patients | Excluded  patients  (n=921) | *P* value |
| --- | --- | --- | --- |
|  | (n=2901) |  |  |
| Age, y | 65.4±12.2 | 67.8±13.2 | <0.001 |
| Men | 1793 (61.8) | 492 (53.4) | <0.001 |
| NIHSS score at admission | 3.0 [1.0, 6.0] | 5.0 [2.0, 15.0] | <0.001 |
| **Risk factors** |  |  |  |
| Hypertension | 2164 (74.6) | 686 (76.5) | 0.946 |
| Diabetes mellitus | 920 (31.7) | 291 (31.6) | 0.947 |
| Hypercholesterolemia | 622 (21.4) | 185 (20.1) | 0.380 |
| Current smoking | 717 (24.7) | 158 (17.2) | <0.001 |
| Congestive heart failure | 119 (4.1) | 102 (11.1) | <0.001 |
| Coronary artery disease | 686 (23.6) | 150 (16.3) | <0.001 |
| Cerebral artery atherosclerosis | 1727 (59.5) | 531 (57.7) | 0.330 |
| Peripheral artery disease | 258 (8.9) | 15 (10.6) | 0.479 |

Data are expressed as means ± standard deviations, medians [interquartile ranges], or numbers (%). NIHSS, National Institutes of Health Stroke Scale.

**Supplementary Table S2.** Correlations between IAD, IAND, ABID, and baPWV in all patients (n=2901).

|  | baPWV, cm/s | |
| --- | --- | --- |
|  | Pearson correlation coefficient | *P* value |
| **Arm BP, mmHg** |  |  |
| IAD | 0.121 | <0.001 |
| **Ankle BP, mmHg** |  |  |
| IAND | 0.207 | <0.001 |
| **ABI** |  |  |
| ABID | 0.139 | <0.001 |

Data were derived from the Pearson's correlation analysis. ABI, ankle brachial index; ABID, ankle brachial index difference; baPWV, brachial ankle pulse wave velocity; BP, blood pressure; IAD, systolic inter-arm blood pressure difference; and IAND, systolic inter-ankle blood pressure difference.

**Supplementary Table S3.** Predictors of short-term outcome.

|  | All patients (n=2901) | | Patients without PAD (n=2643) | |
| --- | --- | --- | --- | --- |
|  | OR (95% CI) | *P* value* | OR (95% CI) | *P* value* |
| **Arm BP, mmHg** |  |  |  |  |
| Diastolic IAD | 0.999 (0.995‒1.004) | 0.769 | 1.000 (0.995‒1.005) | 0.965 |
| Diastolic IAD ≥15 | 1.188 (0.647‒2.181) | 0.578 | 1.435 (0.730‒2.820) | 0.295 |
| **Ankle BP, mmHg** |  |  |  |  |
| Diastolic IAND | 1.043 (1.025‒1.060) | <0.001 | 1.030 (1.005‒1.055) | 0.020 |
| Diastolic IAND ≥15 | 2.226 (1.455‒3.406) | <0.001 | 1.651 (0.893‒3.051) | 0.110 |

Data were derived from the logistic regression analysis. BP, blood pressure; CI, confidence interval; IAD, inter-arm blood pressure difference; IAND, inter-ankle blood pressure difference; NIHSS, National Institutes of Health Stroke Scale; OR, odds ratio; and PAD, peripheral artery disease. *adjusted for sex, age, NIHSS score at admission, hypertension, diabetes mellitus, hypercholesterolemia, current smoking, congestive heart failure, coronary artery disease, cerebral artery atherosclerosis, and stroke subtype.

**Supplementary Table S4**. Predictors of long-term outcome.

|  | All patients (n=2939) | | | |  | Patients without PAD (n=2699) | | | |  |
| --- | --- | --- | --- | --- | --- | --- | --- | --- | --- | --- |
|  | All-cause mortality | | MACE | |  | All-cause mortality | | MACE | | |
|  | HR (95% CI) | *P* value* | HR (95% CI) | *P* value* |  | HR (95% CI) | *P* value* | HR (95% CI) | *P* value* | |
| **Arm BP, mmHg** |  |  |  |  |  |  |  |  |  | |
| Diastolic IAD | 0.999 (0.995‒1.003) | 0.620 | 0.998 (0.994‒1.003) | 0.482 |  | 0.999 (0.993‒1.004) | 0.618 | 0.998 (0.993‒1.004) | 0.538 | |
| Diastolic IAD ≥15 | 1.198 (0.754‒1.902) | 0.444 | 1.188 (0.647‒2.181) | 0.578 |  | 1.149 (0.671‒1.970) | 0.612 | 1.053 (0.647‒1.714) | 0.835 | |
| **Ankle BP, mmHg** |  |  |  |  |  |  |  |  |  | |
| Diastolic IAND | 1.020 (1.008‒1.032) | 0.001 | 1.014 (1.003‒1.025) | 0.012 |  | 1.003 (0.981‒1.024) | 0.819 | 1.004 (0.986‒1.023) | 0.654 | |
| Diastolic IAND ≥15 | 1.199 (0.867‒1.657) | 0.273 | 1.098 (0.807‒1.495) | 0.550 |  | 0.800 (0.457‒1.399) | 0.433 | 0.885 (0.542‒1.444) | 0.624 | |

Data were derived from the cox proportional hazards regression analysis. BP, blood pressure; CI, confidence interval; IAD, inter-arm blood pressure difference; IAND, inter-ankle blood pressure difference; HR, hazard ratio; MACE, major adverse cardiovascular events; NIHSS, National Institutes of Health Stroke Scale; PAD, peripheral artery disease. *adjusted for sex, age, NIHSS score at admission, hypertension, diabetes mellitus, hypercholesterolemia, current smoking, congestive heart failure, coronary artery disease, cerebral artery atherosclerosis, and stroke subtype.
